# Functions of a Convalescent Camp

**Published:** 1918-12

**Authors:** 


**The Functions of a Convalescent Camp.** Major Neff. M. C.

*Purpose* : The function of a convalescent camp is to return the soldier to the front line in fighting trim. In doing this it also relieves the hospital of all soldiers who are intended for future service after convalescence is established, and continues their treatment to the end.

It incidentally simplifies the problem of transportation and aids in finding soldiers who are missing. Many admissions may be absolutely normal men from a physical standpoint, but men who have lost their aggressive spirit. It is the first function of the camp to return men with this spirit renewed, and all schedules and routines are planned with this in view, his physical schedule being regulated by his medical classification and diagnosis. To emphasize further the purpose of the camp and its psychological aspects, it is only necessary to view the attitude of a soldier on admission.

He reflects his mental and physical status. He may be exhilarated by the fact that he has met the enemy, but if he is severely wounded with resulting pain and shock, or gassed, or suffers severe preliminary fatigue, he may recover physically, but the mark of battle is on him and he has the memory of experiences passed through. He may be physically active, mentally alert but morose, apprehensive and dissatisfied. It is the purpose of the camp to remove this memory.

*Admissions* : Detachments provided with a nominal roll, which contains the information of Form 52 temporarily classifying the patients as A, B, or C, are received at the clinic building. They are, as usual, examined for venereal disease, scabies and vermin by the medical officer of the day, and are marched to headquarters by the duty sergeant of the camp, where their field records, clothing slips, and other details are checked, and the detachment is inspected as to its equipment.

After a short lecture on standing orders, daily schedule, Boche spies, etc., by the medical officer of the day, or by the commanding officer, they are divided *pro rata* and marched to their respective platoons. The admission classification is furnished to the platoon clerk, and the soldier reports for duty to his proper class.

*Classification* : The first important point in the success of the routine depends upon the accuracy of the classification from a physical standpoint. The soldier must not be made to do more than he can do with energy and enthusiasm. He must not be whipped beyond his endurance. His physical test must be at first within his easy accomplishment, so he may know and feel reassured that no test will come to him that he cannot master with ease. To this end, the skilled physical and recreational trainer keeps a sharp eye on the new entrant to catch the lagger who is being too severely pushed in order that he may be properly reclassified. Once properly classified, the true spirit of enthusiasm and competition can be rapidly pushed forward.

*Diagnosis* : After admission and temporary classification, all cases are examined carefully and reclassified within one week. On the basis of re-examination the soldiers are changed in grade, and special schedules are instituted for special medical types of case. Special schedules are arranged for cardiac or effort syndromes, joint cases, war-neurosis, convalescent infectious disease and hospitalism. Cardiac and effort syndrome types will be discussed by Lieutenant-Colonel Cohn. Joint conditions have been outlined by Lieutenant-Colonel Goldthwait. I will discuss briefly hospitalism and certain types of pulmonary condition.

*Hospitalism* : With a long stay in hospital a large number of patients develop a lack of independence, with a loss of physical and nervous energy, flabbiness of muscle, a lack of control, and a lessened incentive to duty. Discipline is decidedly interfered with. There is a disinclination to return to work; he tries to secure light and secondary work about the hospital; in other words he digs into an easy job.

*Pulmonary Cases* : At the present time forty per cent. of the admissions are gas cases. Externally these patients may present the physical appearance of healthy soldiers. Their chief incapacity is indicated by cough and expectoration of moderate extent, with or without effort syndrome added, without fever, or signs of serious inflammation in the chest. Serious cases are naturally retained in the hospitals. It is proposed that the lung condition shall receive special attention by postural drainage as soon as complete diagnosis is made. The patient will be instructed and drilled in the carrying out of pulmonary toilet by posture so that his lung secretion may remain below the cough and expectoration level. He will be classified in exercise so that this is accomplished, and drainage will be instituted on schedule every three hours, or with longer intervals, the procedure being introduced into the calisthenics schedule.

By securing early and thorough drainage in these mild cough and expectoration cases, it is hoped to reduce them more quickly and more thoroughly to the non-expectoration stage. This is an experimental procedure, and it is hoped that its results may be as good as in chronic non-tuberculous lung infection.

*Routine and Treatment*: The object that is constantly held in mind is the establishment of the competitive spirit. The treatment in general is exercise by grades, work that interests the soldiers and play that diverts him — plenty of cheer-up, games, competitions with prizes, competitions between battalions, inter-company competitions, interspersed with calisthenics. Good music, concerts and plays are added from the talent which must be furnished by the soldier himself. His time must be fully occupied, he must be kept busy and taught to enter into the light-hearted spirit of the camp, to learn good fellowship over again, to take pride in the American Army, to be a soldier. Special training systems are introduced for special types of medical cases, but a general schedule is held to by all. Rainy days are used for boxing instructions, use of the house-wife, etc.

### Schedule of Exercises for Convalescent Camp.

Cases are admitted on the basis of their referred temporary classification, are reclassified on regular days, and have the following routine :

#### Class "A" and "B"

(Daily with the exception of Saturday and Sunday)

Convalescent Officers below the rank of Major fall in rear of detachment and take physical exercises same as enlisted men.

| | |
|---|---|
| A.M. | |
| 8 : 00-8 : 45 | Calisthenics (O' Grady-Style). |
| 9 : 00-9 : 15 | Hot Tail. |
| 9 : 25-9 : 40 | The men are divided into groups and take part in any of the following games : Three Deep; Leap Frog; Whip to the Gap; Places Change; Jumping the Bag; Crows and Cranes; Giants and Dwarfs; Fox and Geese; Tug of War; Wheelbarrow Race; Soccer Football; Do this; Do that; Struggle; Crawling the Tunnel; Dead Man; Cartwheels, etc. |
| 10-10 : 30 | Jumping exercises, standing, running broad and hop, skip and jump. |
| 10 : 30 | Recall. |
| Saturday : | Cross-country run after calisthenics. |
| Sunday : | Holiday. |
| P.M. | |
| 2 : 00 | Baseball games, races, boxing and football. Dodge the ball, etc. |
| 3 : 30 | Recall. |

CLASS " C "
(Daily except Sunday).
Including Effort-Syndrome and Gassed Cases.

| | |
|---|---|
| A.M. | |
| 8 : 00-8 : 45 | Calisthenics with Class " A " and " B " men. |
| 9 : 00-9 : 45 | Mild relay races; passing the ball to the rear in column, etc. |
| 9 : 30-9 : 40 | Falling exercises for the arms. |
| 10-10 : 30 | Jumping contest with Class " A " and " B " men, except specials. |
| 10 : 50 | Recall special system for special cases. |
| P.M. | |
| 2 : 00-2 : 30 | Indoor baseball or ten pin style relay race, except specials. |
| 2 : 45 - 3 : 00 | Passing the ball. |
| 3 : 30 | Recall. |
| Sunday : | Holiday. |

NOTE. — Before and after each game the men are brought into platoon formation in order that the military feature of the training will not be slighted. Dancing (Highland Fling and Lancers) will be introduced later. Detachments arrive at, and depart from, destination in Military Formation.

For the treatment of war neurosis the patient is employed in trades or mechanics and other camp duties in line with training in civil life, and this is followed by a system of calisthenics. Laggers and shirkers must be disciplined. A mild discipline is a long march without music.

*Re-classification* : Re-classification is carried out once a week. 95 o/o of the cases move up regularly to the exercise grades. If the convalescent period is longer than six weeks, the patient is returned to a disability board for reconsideration.

*Results* : Soldiers usually become cheerful, contented and, except for a wander-lust with a desire to see France, are ready for evacuation from the camp after an average stay of two to six weeks.

At 2 P. M. on the day preceding the evacuation for the Front, the troops are reviewed by the commanding officer, and each soldier is given an opportunity at inspection to report any reason why he should not be evacuated. Evacuation is usually at the week-end in conformity with weekly reports of the medical officers of the section. Conducting officers accompany the troops to their destination. A disability board is appointed from the center which meets as often as necessary to examine soldiers recommended by the medical officer.

*Military Routine* : Garrison schedule is conducted with evening parade and band concert, and adds to the spirit and life of the camp.

*Special Problems* : With a staff of special medical officers for cardiac, pulmonary, and orthopedic conditions, statistical accuracy will be obtained as to the endurance of evacuated types on the fighting line. It is obvious at present that gas cases, particularly those that present effort syndromes, have poor staying power at the front. Length of stay in convalescent camps may be a factor in overcoming this.

Conclusions: 95 o/o of the men are returned in fighting trim. The activities of the convalescent camp make the soldier fit and stout-hearted. Complete this work with a permanent base staff not out of proportion with the work to be done. It is particularly important that over-staffing shall not be done, and that the staffing shall be done by the commanding officer with a thought to the medical and psychological results to be obtained; the important one being the return of a soldier not only in physical trim but mentally agressive.

